# Is Pierre Michon’s *The Eleven* a political novel?

**DOI:** 10.12688/openreseurope.19922.1

**Published:** 2025-05-28

**Authors:** Nenad Ivić

**Affiliations:** 1 University of Zagreb Faculty of Humanities and Social Sciences, Zagreb, City of Zagreb, Croatia

**Keywords:** Pierre Michon, politics, revolution, terror, fiction

## Abstract

Pierre Michon’s *The Eleven* ( *Les Onze*, 2009) is narrated by a cicerone who entertains visitors to the Musée du Louvre and describes the painting of eleven members of the *Comité de salut public* during the French Revolution, which revisits the history of politics at the decisive moment of *la Terreur*. The novel purports to be a commentary on the painting, the people it depicts, the circumstances of its creation and its author. However, the painting is imaginary, as is the quotation from Jules Michelet’s *Histoire de la Révolution française* that legitimises it. The complex interplay of literary traditions and techniques used in the novel defies the banality of a running commentary on political figures and circumstances told in a realist mode to evoke instead the spectacle of telling/making the history/story of politics as a surge of terror, thus revealing the abyss implicit in the performative situation of the protagonist and his audience doubling/mimicking the text and the reader: originary fiction of politics as imaginative re-enactment of the politics of fiction that redeems the Revolution.

        
*La politique fut d’abord l’art d’empêcher les gens de se mêler de ce qui les regarde. A une époque suivante, on y adjoignit l’art de contraindre les gens à décider sur ce qu’ils n’entendent pas.*
              Paul Valéry, *Regards sur le monde actuel* ( 1 on page 947)
        
*Les optimistes écrivent mal*.
              Paul Valéry, *Mauvaises pensées et les autres* ( 1 on page 803)
        
*Faut-il que je me foute de la postérité ! C’est que ma sagesse sait bien que c’est des cons comme nous autres, et pire…*
              Paul Valéry ( 2 on page 1982)
There are for me certain representations that incarnate history, this simultaneously furious and splendid current […]: it is the Victory of Samothrace, at the top of the stairs of the Louvre, wings to the rear, headless, why make a head when it is history, robe flowing in the air, everything in motion, one can say that *The Eleven*, that is eleven times the Victory of Samothrace. (Michon in 3 on page 50)

It seems that a diversion, several detours via antiquity, literature, politics and language, all the garb that flows in the air, are in some way justified, although the consensus of critics places Pierre Michon firmly in the twentieth and nineteenth centuries.

“History, in all meanings of the word, is above all an essential category in the work of Pierre Michon”; his writing aims to “bring anonymous, infamous heroes back to life” and revive “great figures of the history of literature and the visual arts, or illustrious figures of Great History” ( 4 on page 93–94 *). The Eleven* is a novel about great history; it deals with the founding moment of modernity, the French Revolution, and some of its illustrious or infamous figures, such as Maximilien Robespierre and the members of the Committee of Public Safety ( *Comité de salut public*), the perpetrators of the Terror ( *la Terreur*):

Can you see them, Sir? All eleven of them, from left to right: Billaud, Carnot, Prieur, Prieur, Couthon, Robespierre, Collot, Barère, Lindet, Saint-Just, Saint-André. Unchanging and erect. The Commissioners. The Great Committee of the Great Terror. Four point thirty by three meters, a bit less than three. The Ventôse painting. So improbable, the painting that had every reason not to be, that so well could, should not have been, that standing before it we shudder to think that it might not have been, we appreciate the extraordinary luck of History and of Corentin.
[…]
Can you see them? It is hard to see them all in one glance now, with those reflections from the glass behind which they’ve been placed in the Louvre. Proof against bullets, proof against the breath of ten thousand people from all over the world who look at them each day.
But there they are. Unchanging and erect.
And here is their author. ( 5 on page 31–32; 6 on page 43–44)

*Ecce pictura*. Suddenly, after a few pages, it is there and the reader can read it: the painting, the image. An epiphany: “We are here before it. See how they change when you move to the left” ( 5 on page 95; 6 on page 129). Is the movement to the left a political statement encoded in the change of perspective?

The painting of eleven men who preferred to be seen as “figures of Homer rather than the combination of Lycurgus and Alcibiades by which we know them” ( 5 on page 38; 6 on page 52). The image put into words. Before Christianity, “the Greeks defined a mode of expression they called *ekphrasis,* which can be translated as ‘putting into words’” ( 7 on page 27). It goes back to the famous shield of Achilles in the *Iliad*, which represents the main protagonists in the greatest conflagration of Antiquity, at least for those familiar with ancient philology. The *Iliad* is an epic, it depicts the suffering of the Greeks, and historians, when they began to write history, capitalised on this: Guided by the epic principle, they recounted the great deeds of men (see 8 on page 71–72). They described the suffering, the invasion of the Other, the hostility, the enemy and the war and let the political happen: One must “start from hostility in order to arrive at the political” ( 9 on page 152). These revolutionaries liked to see themselves in Greek garb; Lycurgus and Alcibiades, authoritarian legislator and populist politician respectively, and Athens and Sparta represented a real alternative for them as they debated the form of their republic. They are a consequence of Homer: politics springs from epic poetry, which describes hostility, war and suffering.

Hostility against whom? Who is at war with whom? Alcibiades against Lycurgus, revolutionaries against counter-revolutionaries, tyrants against the people? Who is the enemy? The aristocrats of the eighteenth century, the French citizens or one of the ten thousand people from all over the world who see them every day in the museum? And the person who is supposedly going to fire a gun, that unknown member of the public against whom the painting is covered with glass, “proof against bullets” ( 5 on page 44; 6 on page 32), does he have something against the painting, against what it represents, or against the fact that it is in the museum? And what is this something? Exhibitionism, politics, insanity? All these questions point to the impossibility of speaking of a specific ‘context’, of clearly outlining a historical context, a space-time of *The Eleven*, despite its firm chronological anchor: the French Revolution. If access to the political comes from hostility, and if hostility comes from history or stories, then what is history? On the one hand, the headless context of the Revolution titillates between past and present, artefact and fact, attitude and action; on the other hand, the headless histories of the Revolution ( 10 on page 15) “are their own reference and each of them determines its enemies” ( 11 on page 83).

The words are those of praise. They describe a masterpiece. *Ekphrasis*, even unfinished, never really completed, as in *The Eleven* ( 12 on page 143), is also *epideixis*: speech, discourse, eulogy, going back to the one-man show of Gorgias, who absolved Helen of guilt for the Trojan War. The painting, conjured out of nothing, as it were, by the power of words, suddenly becomes an object; the “phantom committee” is transformed “into a real committee, theatrically real” ( 5 on page 72; 6 on page 98), and this theatrically real object “becomes, in the eyes of all, objectively the effect of the omnipotence of the Logos” ( 13 on page 132). The affirmation of reality, of ‘this has happened’, characteristic of the discourse of history ( 14 on page 139), is a performance: Someone, an ambiguous omniscient narrator, cicerone or art historian, a combination of lecturer and smooth talker or media presenter paired with philosopher-journalist, “droning on […] with [his] vague theories” ( 5 on page 50; 6 on page 69), praises the painting in front of someone singled out from the usual crowd, and tells of its creation: “It’s a political commission, that goes without saying: so let us lower ourselves for a moment and talk politics” ( 5 on page 67; 6 on page 91). If this reality effect achieved through language, if this figment of imagination turned real is the result of praise, and “the reason why praise is at the same time, even before anything else, praise of the Logos” ( 13 on page 132), then a fundamental question arises between the narrator and the narratee that goes beyond hearsay: a question about the Logos in politics and the politics of the Logos, about the guilt and exoneration of the Logos.

The performance does not take place in a kind of assembly, but in a museum.

The setting, the staircase, the hall and the museum Le Louvre are obviously real, as is confirmed by the breath of ten thousand people from all over the world who come to look every day. The painting and its author, Corentin, on the other hand, are invented, not in the sense of “some degree of imaginative creation”, but in the ancient, rhetorical sense of the word: “with the implied theory being that this is somehow already there, though latent, and does not have to be made up as a mere figment of the imagination” ( 15, qtd in 16 on page 289). The subject is familiar: The painting depicts eleven members of the Committee of Public Safety, the main protagonists of *la Terreur*, the *crux* or *locus desperatus* of the French Revolution. Following an axiological, epic principle, *The Eleven* chooses a historical moment that produced great deeds and great suffering, the Revolution and the Terror, and praises or blames history in a performance that describes the image of its perpetrators. The choice of subject, “a great subject known by all, legendary, a subject of the community that can be shared” ( 17 on page 47), concerns the entire body politic, the foundations of the Republic. The elaboration of the subject, the writing or *ekphrasis* that turns the subject into an object, “marks, beyond the ontology/logology difference, the moment of ‘political invention’ that passes from the community in the values of language through the meaning of words and metaphors […] to the creation of new values” ( 13 on page 132–133). The invention of the image in literature presumably represents a political invention beyond the difference between reality and fiction.

Are these new values, however political they may be, due to the power of the particular constellation of words we call the novel? The title says nothing about it; there is no genre designation, although the work is usually called a novel, a historical novel ( *roman historique*), because of its generally recognised historical theme. Critics claim that, in Michon’s corpus, *The Eleven* “comes closest to the genre of the novel” ( 12 on page 185). Michon himself says that he writes “short novels, condensed, tightened, degreased” ( 17 on page 24). But what does it mean to condense, tighten and degrease a false genre, a “non-generic genre that has never stopped travelling […], the genre of what has no genre” ( 18 on page 51)? The impossibility of defining a context, of stabilising and saturating it under the concept of history, is echoed in the impossibility of defining the genre, of stabilising and saturating *The Eleven* under the concept of the novel. The history of the genre and the genre of history are a sea of differentiated meanings, and *The Eleven*, with its “transverse writing that invents itself diagonally to the epochs” ( 12 on page 247), can be compared to a ship of words constantly navigating between the stream of the living and the stream of the dead, laden with “universal freight that makes beautiful paintings” ( 5 on page 45; 6 on page 62), full of migrants, false papers and forgers; it represents the falsity of history, in the truest sense of the word, travelling on the wave of history and the tide of stories, from the present moment to the distant past and back again.

This charge of praise and blame, the sheer force with which it is delivered by a verbose and sceptical psychopomp, drives a wedge of *dissensus* among the readers, who belong to a community tired of “political quibbling” ( 5 on page 79; 6 on page 106), largely unaware of or disdainful of the sceptical questioning that opened up the boundary between history and fiction ( 19 on page 7) and accustomed, since the 1980s, to “an ‘ethical’ mobilisation (in favour of victims of starvation or of racism, for example) which remain[s] the last political rallying point, focused on specific issues and highly mediatised”, a kind of “humanitarian moralism” ( 20 on page 314), characterised by “the achieved uniformity of viewpoints” ( 17 on page 17) and cultivated by the *nouveaux philosophes* (Alain Finkielkraut, André Glucksmann, Pascal Bruckner, Bernard-Henri Lévy) that is still common in today’s political life. In the nineteenth century, “in order to make, to think or even to dream the Republic, the Revolution must be forgotten” ( 21 on page 895); in the twentieth century, after the traumatic experiences of Russia and China and especially after the events of 1968, for the *nouveaux philosophes*, “the revolution”, stained and damaged, “must be declared impossible, forever” ( 22 on page 3).

*The Eleven* is not a straightforward historical novel like Victor Hugo’s *Ninety-Three*, for example, with its vast *tableaux* depicting political milieus, teleologically orientated plots and imposing, monumental figures adorned with consuming passions and embodying political ideas. This is not an authorised version of the French Revolution, as Hugo created it for generations of budding French citizens as part of civic lessons in secondary schools to “enable the French to love *all* their history”, Revolution and Terror included ( 21 on page 941). Rather, it presupposes a clear break with the Romantic, Hugonian tradition of the historical novel and its “immanent meta-politic that makes the mute speech of things the truth of the loud speech of fighters” ( 23 on page 69). As a narrator in Michon’s *Corps du roi* says when talking about Hugo’s poetry: “I defeated these verses. I was a free man” ( 24 on page 97), *The Eleven* repeats the gesture – the gesture in literature becomes a gesture of literature – and rejects this romantic fatherhood, symbolised by the towering figure of Hugo, albeit in a slightly different way.

Michon does not reject the poetic *souffle* of Hugo (or of Racine, Villon, Baudelaire or Rimbaud): Michon “must constantly remind himself of the rhythm of the verse in order to converse with the dead” ( 4 on page 99–100). His conversation bears certain similarities to the historiographical operation of Michel de Certeau, for whom “in the West, during the last four centuries, making history goes back to writing” ( 25 on page 12) in order to produce a “kind of thanatography, for to write history is to entertain oneself and to erect the tomb for the dead” ( 26 on page 247) in both meanings of the word *tombeau*: the actual burial place and a poetic form, a tribute to one of them by one or more writers, usually in verse. Michon’s writing is exquisite, thoroughly poetic, similar to the ancient history that Quintilian would call a *carmen solutum*, a prose poem or, more aptly, a poem set free ( *Institutio oratoria*, 1.10.31). He knows that “you need imagination to close the gap between the present and the past” ( 16 on page 75) and recognises the need for poetic power to storm the barrier of contemporary presentism and close that gap. For him, as for Quintilian, history is *proxima poetis*, close to poetry in a particular sense: The poetic *souffle* frees the novel’s prose from the influence of ‘the universal reportage’ (Mallarmé) of historiography and its vulgate.

In *The Eleven*, the freedom of poetic discourse results from the rejection of a certain kind of historical imagination that is necessary to close this gap. It is the historical imagination that results from a proximity or closeness of literary, novelistic writing and historiography. Michon rejects the use of this proximity and closure forged in the nineteenth century by a connection between literature and historiography, when the romantic historical novel, e. g. by Walter Scott, served as a model or ideal of historiography (Augustin Thierry, Jules Michelet), which, in turn, served as a model or ideal of literary, realistic writing “as in a reflection, superimposed” ( 5 on page 44; 6 on page 60). For Michon, the liberation of literary discourse from the heroic model of science ( 27 on page 161) and the use of historiographical writing as the degree zero of the historical novel seems to be the precondition for his own poetic *souffle*. By reinvigorating the communion in the values of language, he hopes to create a new value of history.

Through the magical act of writing, the *scaena* of *The Eleven* becomes obscene, an *ob-scaena* of sedimented interpretations of the French Revolution and ready-made objects to be embellished by literature, implying the critique of the historiographical methods with which it was treated. In a sense, Michon’s ‘counter-narrative’ of the Terror, in breaking with the bicentenary commemoration of the French Revolution ( 12 on page 107), mimics François Furet’s gesture in his *Penser la révolution française* (1978, see 28), presenting “1789 as a moment of ‘historical deviation’ in order to free himself from the ‘revolutionary catechism’ of the Marxist historian Albert Soboul” and thus initiate “a historiographical rupture” ( 26 on page 250–251). But unlike the rabidly anti-Marxist Furet, whose “reductive thematics of mythology nevertheless place interpretations in the space of the unreal”, Michon’s *The Eleven* can be seen not as a polemical stance but as a complement to Furet, a descent into political mythology and its anthropological origins (see 29 on page 135). If we were to look for affinities or models in historiography, *The Eleven* could be compared to a fictional *microstoria* or microhistory. Microhistory, as Natalie Zemon Davis maintained, requires “details, evidence, and the ambition of *histoire totale*” ( 30 on page 69), as in Carlo Ginzburg’s *Indagini su Piero* (1981, see 31), which unfolds the story of a great painter, Piero della Francesca, and his commissioned masterpiece *La flagellazione di Cristo* with its depiction of great political figures implicated in major contemporary events. It painstakingly reveals the chiselled specifics of the commission and its protagonists, embedded in the great wall of history, “the ever enigmatic wall of eleven men upon which History is perched” ( 5 on page 121; 6 on page 127). This time it is not about the archetypal Roman emperor in Byzantine robes John VIII Palaiologos (Ἰωάννης Παλαιολόγος), the mythology of *imperium* and the unity of the Church, but about the archetypal bloody revolutionary Robespierre, the mythology of Revolution and the unity of humanity.

The roots of this gesture lie much deeper, in the personality of the implied author alluded to and in the troubled civic memory of the founding of the Republic. In his other works, Michon “deploys a certain hermeneutic of the self; he metabolizes the absence of [his] father by realising a unique form of mourning work through literary writing” ( 26 on page 247). The work of mourning amounts to a “block of prose” – Michon is not fond of the word novel – exhibiting repetition: One absence or vacancy after another, preceded by acts of execution and “naked repetition of the origin, which, on the contrary, does not exclude the fact that nakedness is revealed only in repetition and in history” ( 32 on page 49) in the meticulously told story of the protagonist’s father and in the history of the fathers of the Revolution, all failed writers, except Robespierre, “who needs no comment” ( 5 on page 39; 6 on page 54: in French: “ *qu’on ne commente pas*” [who should not be commented on]). “The artist as son, that is the rule after 1789, that is the eternal romanticism that we have not yet abandoned; we do not stop killing the ghostly father who was seriously slain long ago, no doubt in January 1793, under the great cleaver of the sons, in the square of the Revolution, that we now call Concord Square, seriously” ( 17 on page 54). The hermeneutics of the self, the self as author and budding literary failure and the self as political animal, ζῷον πολιτιϰόν, repeats its own origin in the narrative, in history, in the stories, revealing a nakedness of a parricide/regicide not to be commented on.

The beginning of the novel sets the tone with a portrait of the painter: “He was not tall, unobtrusive, but he held your attention to himself with his feverish silence, his dark cheer, his alternately arrogant and oblique manner – grim, as they said” ( 5 on page 9; 6 on page 13). It is the liberation from a certain kind of historiography, from “two centuries of exhaustive efforts to understand the reason for [the commission of the painting]” ( 5 on page 67; 6 on page 91), from the ominous historical ‘as they said’ that runs as a *basso continuo* in many forms throughout the text, masking moments of execution with familiar, casual language ( 4 on page 101). To free oneself from ‘as they said’, from the paternalism of universal reportage, is to move through language in another arrogant and oblique way, to restore the feverish silence and dark exultation of history, to breathe life into it and open up a sombre space “between the subjects to come”: “This in-between, the space of the other, is the site of radical disfigurement” ( 32 on page 76). The disfigurement of history by story, of story by history, opens up the possibility of telling stories and histories about the same points of history and politics that differ from the accepted, uniformed history. From the outset, *The Eleven* introduces a difference into the universal republican discourse on Revolution.

“Corentin was the son of a man who chose literature, who sacrificed everything to it and was broken by it. A man to whom letters gave in turn, hope, spite, and shame” ( 5 on page 38; 6 on page 50–51); “he put the figure of his father in the form of the eleven murderers of the king, the Father of the nation – the eleven parricides, as the king’s murderers were then called” ( 5 on page 41; 6 on page 54). The metaphorical murder of the father by literature, which repeats the literal murder of the king, the father of the nation, is in turn repeated by the literary failure of the protagonists of the Terror, “powerful broken-down machines, widowers of literary glory” ( 5 on page 41; 6 on page 56). The repetition transforms the mere appearance of the text into a revolutionary gesture and connects it directly not with later revolutions, but precisely with the singularity of the French Revolution. “The French Revolution is radically different from the ideal revolutions that claim to be its successors”: It created “a deliberative space”; unlike other revolutions, English or American, “it began with the affirmation of rights”, “it spoke of universality” ( 7 on pages 252, 256–257). Hence the sombre jubilation and feverish silence over the murder of the universal and particular father, the parricide and regicide, his fleeting presence and haunting absence. History can be found in the figure of the father, who must be rendered headless and disfigured in order to clear the space for reflection in which universality can be affirmed and realised as a possible impossibility through the universal rights of literature. The failure of literature is always the gain of literature.

Poetic and *poethic*, uniting poetry and ethics, Michon’s phrasing in *The Eleven* can be seen as a variant of what Erich Auerbach called the Tacitean in *Mimesis*. Not only because ancient historiography, and Tacitus in particular, can by and large be considered a good example of what is generally accepted as the political novel, or as Cicero elegantly put it, “ *concessum est rhetoribus ementiri in historiis, ut aliquid dicere possint argutius*” ( *Brutus*, 42), it is permissible for rhetors (historians, writers) to lie (fictionalise) in histories in order to say something more subtle. The subtlety results from Tacitus’s handling of language. “In Tacitus, the sombreness and weightiness of the historical style, reinforced by the sombreness of the events he reports, is charged with sensory perception. Time and again it is there, evoked by the suggestive power of horrible happenings but only to be quickly repressed again by the refined and pointed brevity of the style, which will not allow for such outbursts to prevail” ( 33 on page 51). Moreover, Tacitus had the ability to forge a language that suited his characters, for example in the case of Tiberius, whose “style of discourse [was] more congenial as none other to Cornelius Tacitus” ( 34 on page 319). Tacitus describes Tiberius’s way of speaking as *suspensa semper et obscura verba* ( *Annales* 1.11): using always ambiguous and obscure words.

“Ambivalence is at the heart of my personal mythology”, says Michon ( 17 on page 231). Jean-Pierre Richard interprets it as “hesitant rhetoric” ( 12 on page 85): His phrasing is obscure because only the alternative discourse of poetic history in which they are set sheds light on them. The meaning of the words is literally suspended as if they were bound on the one hand to literary and artistic tradition, modernity and the avant-garde – Michon’s text is a “palimpsest” ( 4 on page), a “metonymy of the library” ( 12 on page 31) – and on the other to the tradition of historiographical interpretation, to ‘as they said’ and hearsay: “His historical and sociological analysis undoes the magic [of his own poetic phrasing] and brings it back to its conditions of production, to its other side” ( 4 on page 97). This suspense of words goes even further: The very difficulty of isolating bits of reality and science from bursts of fantasy, of determining their origins and juxtaposing them, as models or paraphrases, to distil something that could be called political, constitutes the entrance to the deliberative space of *The Eleven,* where this descent into the political mythology of Revolution can be achieved. As in poetry, it is precisely this difficulty that opens up access: “Suddenly, with ease, we find ourselves accessing, i. e. in the absolute difficulty, ‘sublime’ and ‘touching’” ( 35 on page 10) of Revolution, art and terror. “There is a certain stupidity or clumsiness in literature that thinks other than through metaphor”, Michon claims ( 17 on page 60). As poetry, *The Eleven* is not about problems, but about difficulties ( 35 on page 10) – difficulties of metaphor and in metaphor, in literature and of literature, difficulties in history and difficulties of history: “Because they tell stories, Sir, and so do we” ( 5 on page 23; 6 on page 30).

What is supposed to pose a problem or mimic a sociological analysis, “the drab history and theory” ( 5 on page 79; 6 on page 106), is relegated to the realm of stories and dismissed: “I wonder, Sir, if it is really useful to tell you all this, these family histories and these noble ancestries, so prized by our era; if it is necessary to go back so far, to these pale existences that are only hearsay after all, hypothetical causes” ( 5 on page 22; 6 on page 29). On the contrary, what is supposed to be fictional, the painting, is asserted and recognised as real: “We have had the indubitable existence of *The Eleven*, that definite block of existence, irrefutable, unchanging, the solid effect, that does perfectly well without causes and that would do perfectly well, too, without my commentary” ( 5 on page 22; 6 on page 29). *Figura etymologica* perhaps: Comment, *commentarium*, is inseparable from *commentum*, an invention as fantasy, fiction, lie; these two words are etymologically linked in Latin. In *The Eleven* there are no clear boundaries between reality and art, between document and fiction, history and writing, political fact and commentary on it (one of the definitions of the political novel). “Every statement contains its counter-statement”, Michon emphasises ( 17 on page 188): Everything is undermined and contested, even the form of the commentary itself; “the series” of statements that make up the narrative of *The Eleven*’s story, the painting and the novel, “hangs on the artifice of references”, on hearsay, on an imaginary Michelet, and “constantly eludes the exorbitant presumption of origin” ( 11 on page 64). The text takes its autoreferentiality to the extreme.

The recourse to history serves to “liquidate reality” ( 17 on page 232). The extreme touches its opposite. Consequently, they are to be taken *en bloc* (hence the block of prose), all at once, so to speak, as a superfluous utterance, a futile exercise of contemporary literature haunted by its impotence and obsolescence. Solidified in a block of prose, it is transformed into a *notice assommante* ( 5 on page 69, not translated into English), a knockout notice that, “through the smallest and most secret (invisible) body, performs the most divine works” (ὃς σμικροτάτῳ σώματι καὶ ἀφανεστάτῳ θειότατα ἔργα ἀποτελεῖ: Gorgias, *Helenae Encomium*, 8 Donadi). Gorgias is echoed by Michon’s “violent and voluntary nothingness that sustains the figures, that makes them standing” ( 17 on page 140). Like Greek tragedy, the phrasing of the prose block “uses legends and emotions to create a deception in which the deceiver is more honest than the non-deceiver and the deceived is wiser than the non-deceived” (Gorgias, DK76 23B). It would be better to translate the Greek ὁ απατήσας as ‘the one who creates an illusion’, with connotations of pleasure, seduction, deception, trick, cunning and trickery. This is the divine work, the magical feat that the ambiguous narrator accomplishes through *The Eleven* and its standing, theatrically real figures of the eleven members of the Committee of Public Safety: not the production or reappraisal of historical truth, but the affirmation of “the violence of the role of fiction, which is constitutive of the narrative […], the fixation of fiction, the decided, desired and assumed fabrication of the common history” ( 13 on page 190).

The fixation of fiction in the image makes stories the measure of stories, fiction the measure of the truth and falsity of reality. If the event is a summary of the narrative and the narrative a “very detailed description, or mis-description, of the event” ( 36 on page 73–74), then fiction, which is both description and misdescription, proves to be a revenant, a spectre that constantly haunts the reality and history of the event. Fiction thus becomes “the trope [Greek: τρόπος; Latin: *tropus* and *modus*; French: *écart*; German: *Wendung*] of the better, in the sense of what is ‘more useful’, that the truth is invited to take, or, it [becomes] the point of impact on the truth, of that ‘beautifully politicise’ of Aristotle” ( 13 on page 190) against history, in which “the desire for truth is the historian’s weak point” ( 37 on page 107). Though possible, the English translation, ‘statesmanlike action’, misses the point: πολιτεύσασθαι καλῶς, literally ‘beautifully politicise’, refers to the liquidation of reality, just as Archinos liquidated those who remembered the horrors of internal war (Aristotle, *Athenaion Politeia* 40.2). We return to thanatography, to the sovereign, purifying power of death. “This is Lascaux, Sir. The forces. The powers. The Commissioners. And the powers, in the language of Michelet, are called History” ( 5 on page 97; 6 on page 132). Just as the genealogy or archaeology of the painting returns at the end to prehistory, to the primaeval forces of predation and the apotropaic power of the Lascaux paintings, the genealogy or archaeology of politics, evoked by the imaginary, shifting, unsaturated context of the novel, returns to its roots, ancient Greece, philosophy and sophistic. To close the circle, the dissolution of reality requires a huge anthropological diversion, a dramatization of primitivity in the sense of Georges Bataille and André Leroi-Gourhan.

“In *The Order of Things*, Michel Foucault articulates discourses on images. The book begins with a commentary on *Las Meninas*; it is more than a rhetorical device to captivate the reader. The text shows that a discursive dispositif, an apparatus or construction, determines a possible painting” ( 7 on page 26). *The Eleven* re-enacts this determination: Life, art and history, “all those moving, living forms mean nothing more than this, tossed out to end up in a painting that repudiates them, exalts them, bludgeons them, weeps for that devastation and inordinately delights in it, eleven times, through eleven stations of the flesh, eleven stations of wool, silk, felt, eleven forms of men; all that makes sense and is spelled out clearly only in the page of darkness, *The Eleven*” ( 5 on page 13; 6 on page 18). The moving forms of life, history and art determine the side of darkness in the painting.

At the same time, Corentin’s painting, via words, “puts into image that discursive dispositif, apparatus or construction” ( 7 on page 26):

So perhaps this is what you think: behind the glass are eleven appearances of Corentin de la Marche. Corentin de la Marche eleven times. The father and his vocation, his alibi, eleven times. Eleven times the hand with the pen, the author – but the uncertain, lost Limousin author. All of them the lost offspring of literature, one and indivisible: for they loved glory, the idea of glory, above all else, their presence behind the glass attests to it; and pure glory, in those times as in others, came through literature, which was the occupation of men” ( 5 on page 37; 6 on page 51–52).

“The painting of darkness” ( 5 on page 19; 6 on page 43) puts into image the deadly dispositif of literature.

In other words, a *dispositif jacobin* determines a “force of lightning” of the painting, which, in turn, places “the spearheads” of that *dispositif* in the picture (translated as ‘Jacobean action plan’ ( 5 on page 72; 6 on page 98). “Pale existences” and “hypothetical causes” are transformed into a “block of existence, irrefutable, unchanging, the solid effect that does perfectly well without causes” ( 5 on page 22; 6 on page 29), and this irrefutable block of prose transforms the paleness into (rhetorical) colour and the hypotheses into reality. An alchemist would call these recursive transfigurations *magnum opus*. “We stay” in front of this imaginative re-enactment of darkness transfigured into light, “heads raised, listening to [the Sirens’] circular song as if it were the inextricable story of the world itself that they were revealing to us” ( 5 on page 22; 6 on page 30). What the text alludes to as the Sirens’ song is the deadly call of reality and universal reporting masquerading as history and literature. *The Eleven*, on the other hand, stretches out the rope to bind the implied reader and the narratee to the mast of her word-ship, as Odysseus did when he faced the Sirens, to prevent them from succumbing to reality.

In *The Order of Things*, the *ekphrasis* of Velásquez’s the painting *Las Meninas* establishes the order of visibility and legibility of things; in *The Eleven*, the *ekphrasis* of the painting of eleven members of the Committee of Public Safety establishes the order of visibility and legibility of history and, in particular, the order of interpretation of the Revolution. Foucault himself described *The Order of Things* as a “pure and simple fiction” ( 38 on page 619). “It is a novel, but I did not invent it, it is the relation of our epoch, its epistemological configuration and all this mass of statements” ( 38 on page 619). The fiction of *The Eleven*, its reality effect, is invented by the relationship established at the moment of the commemoration of the Terror between the imaginary configuration of works of art in a museum, Tiepolo and Caravaggio grafted onto David and Géricault, and the mass of statements about history, Furet’s revisionist vulgate grafted onto the romantic, idealising Michelet.

The reader is forced to see the painting put into words, but not to hear the words; when he hears the words, he cannot see the painting because of “the complaining, murdering packs of the eternal, barking plebeians: and through all that barking, no one heard anything anymore” ( 5 on page 69; 6 on page 94). The “page of darkness” and the “painting of darkness” silence each other. They are incompatible. But the painting, which simultaneously misses and exceeds, obscures and reveals the knowledge of the Revolution and its *corsi e ricorsi*, also indicates its vanishing point, a “rabbit hole when the ferret is released, except that here, all are both ferret and rabbit for all the others” ( 5 on page 67–68; 6 on page 91–92). This animal metaphor, the rabbit hole, is a blind spot that reveals the nature of the *crux*: It points to the original connection between Revolution and terror, to the original darkness in which Revolution and terror are indistinguishable. One critic hastens to conclude: The eleven members of the Committee can rightly be regarded as “criminals because they murdered the principle of legitimacy, which is a basis of people’s political existence beyond legality. They have committed democide” ( 39 on page 80). This is the original sin of human history: “Perhaps what Michelet [the author of *Le Peuple*] saw at the end of the Flore pavilion was History in person, in eleven persons – in terror, because History is pure terror” ( 5 on page 94; 6 on page 127). “The eleven living men are History in action, at the height of the act of terror and of glory, that is the basis of History – the real presence of History” ( 5 on page 95; 6 on page 129). Glory and suffering: the epic principle of the conception of history returns with a vengeance. Revolution, all revolutions, are inherently tainted.

Terror plays a major role in the mythology of revolutions. “Subsequent revolutions recalled what may be called the axiom of fear and trembling. They subjected themselves to the test of this axiom. Each one failed” ( 7 on page 52). With the same gesture, the emergence of the painting, an impossible thing, becomes indistinguishable from the emergence of history, and the interpretation of the French Revolution as an archetype of future revolutions merges with history as the story of the prose block called *The Eleven*. Just as the painting of terror deprives history of words, revolutions necessarily deprive people of the opportunity to speak in a “short narrative that captivates the reader, prohibits multiple readings, deprives him of freedom and casts a spell over him” ( 17 on page 25). Revolution is Terror, once narrated, it is necessarily transformed into the silent image of terror: Literature about Terror turns into the terror of literature. *The Eleven*, the narrative that protests against the horrors of Terror, reveals this protest thanks to the terror of the narrative.

But “[h]istory [as history and as story] has a pocket for luck in its belt, a special purse to pay for impossible things” ( 5 on page 32; 6 on page 44). “Billaud, Carnot, Prieur, Prieur, Couthon, Robespierre, Collot, Barère, Lindet, Saint-Just, Saint-André”, all those proper names, which are repeated *ad nauseam* in *The Eleven*, are “in this particular context merely an artifice”: They give us “a finger to point with, in other words, to pass surreptitiously from the space where one ‘speaks about history’ to the space where one looks at the image of it; […] in other words, to fold one over the other as though they were equivalents. But if one wishes to keep the relation of language to vision open, if one wishes to treat their incompatibility as a starting point for speech instead of obstacle to be avoided, so as to stay as close as possible to both, then one must erase those proper names and preserve the infinity of the task” ( 40 on page 9). It is the infinity of the task of narration, of differentiation and nuance, opened up in the deliberative, narrative space of the French Revolution. “If the French Revolution touches the Real, it is not through execution, not through terror, but through discourse, not through versed blood, but through words” ( 7 on page 244). The change we are supposed to see moving to the left side of the imaginary painting is indeed a political statement: It concerns the solution of continuity between Revolution and Terror. The epistemological configuration of revolutionary imagery is exploded and the axiom of fear and trembling becomes obsolete. There is no necessary connection between the deliberative space opened up by the French Revolution and Terror forged by the image of it. The stain of blood is removed.

The deceiver, the ambiguous narrator, a distant caricature of the *nouveaux philosophes* who dominated the French intellectual scene in the last quarter of the twentieth century, that puts the Terror into the image of history, is indeed, precisely because of the inversion and carnivalization implied by his hanging, obscure and deceptive words, more honest than the non-deceived implied readers, a community that commemorates the bicentenary of the Terror, those who, clinging to grand concepts and clear-cut dualisms, firmly believe that history is nothing but terror. When the low talk of the politics of Terror is reduced to the perennial presence of human nature, the high talk of Revolution insidiously becomes an attempt of sublation of its history. One could compare History to the headless movement of the Victory of Samothrace, but the Revolution, beautifully politicised in the Aristotelian sense eleven times in *The Eleven* and laid to unrest in the *tombeau* of literature’s flowing robes, resurrects, if not teleology, for it is always about that pocket of luck that the Greeks called καιρός, then its triumphant, thinking head.

The owl of Minerva takes its flight only when the shades of the night are gathering: “What has become of the night, Sir?” ( 5 on page 77; 6 on page 103), asks the cicerone.

## Ethics and consent

Ethical approval and consent were not required.

## Data Availability

No data associated with this article.
