# Supplementary material for: Is Pierre Michon’s The Eleven a political novel?
Source: Open Res Eur. 2025 May 28;5:144. [Version 1] doi: 10.12688/openreseurope.19922.1 (PMC12398675; doi:10.12688/openreseurope.19922.1)
Supplement: Peer review history

# Reviewer report: Reviewer response for version 1

**DOI:** 10.21956/openreseurope.21550.r55244

**Published:** 2025-08-30

**Authors:** Carol Colatrella

**Affiliations:** 1 Georgia Institute of Technology, Atlanta, Georgia, USA

Ivic's essay thoughtfully considers the ways in which Pierre Michon's novel *Les Onze* represents persons and issues related to the period of the Terror following the Revolution of 1789. The author examines Michon's narrative about a fictional artist who produced a fictional painting depicting eleven ruthless male members of the Committee on Public Safety as "history in action" (7), considering how historical narratives, including the painting, the fictional explanation that the historian Michelet writes, and by extension the novel itself "misses and exceeds" (8) in its representations of violent political actors as heroes who are also monsters. For Ivic, "Terror plays a major role in the mythology of revolutions" (8), making a political statement linking the French Revolution and the Terror. The essay helpfully connects a discussion of ekphrasis as a literary device to the analysis of "Las Meninas" in Foucault's *The Order of Things *and acknowledges Michon's claims about the difficulties of metaphor in telling stories, concluding that *Les Onze* politicizes history in presenting events and actors in literary form.

If any, are all the source data and materials underlying the results available?

Yes

Is the work original in terms of material and argument?

Yes

Is the work clearly and cogently presented?

Yes

Is the argument persuasive and supported by evidence?

Yes

Does the research article contribute to the cultural, historical, social understanding of the field?

Yes

Does it sufficiently engage with relevant methodologies and secondary literature on the topic?

Yes

Reviewer Expertise:

Comparative literature, including the study of narrative theory about history and the novel as genre and representations of democracy in US, French, and British fiction

I confirm that I have read this submission and believe that I have an appropriate level of expertise to confirm that it is of an acceptable scientific standard.

# Reviewer report: Reviewer response for version 1

**DOI:** 10.21956/openreseurope.21550.r55708

**Published:** 2025-08-11

**Authors:** Klevis Kolasi

**Affiliations:** 1 Ankara University, Ankara, Turkey

**Overall Evaluation**

 Nenad Ivić’s article is a highly sophisticated and theoretically ambitious reading of *The Eleven* by Pierre Michon. It offers a nuanced exploration of how Michon’s novel engages the intersection between literary theory, historiography, and the politics of revolutionary memory. Eschewing traditional understandings of the political novel as ideological commentary or realist allegory, Ivić argues instead that *The Eleven* enacts its politicality through meta-historical disruption, aesthetic mediation, and the formal dismantling of narrative historiography.

 Deploying a dense intertextual framework—particularly Foucault’s *The Order of Things* and classical notions of ekphrasis—Ivić interprets *The Eleven* as a dispositif, a performative apparatus through which the novel reveals the constructedness of historical meaning. The central conceit of the fictional painting functions not only as a literary object but as a metaphor for the epistemological instability of revolutionary representation, where the line between fiction and historiography collapses.

 Despite these significant intellectual strengths, the article’s impact is limited by three interlocking challenges:

1. Stylistic opacity and rhetorical overcomplexity, which obscure the author’s critical insights;
2. A lack of methodological clarity, particularly in how the article frames its interpretive approach;
3. Insufficient engagement with key historiographical and ideological debates, particularly those concerning poststructuralist theories of history and the political ethics of representing revolution and terror.

Given the article’s significant potential, I recommend “Approved with Reservations,” subject to substantial revision of style, structure, theoretical clarity, and political contextualization.

**Assessment According to ORE Humanities Criteria**

 1. Is the work original in terms of material and argument?

 Yes. The article makes an original and theoretically rich contribution, arguing that *The Eleven* is political not through ideological positioning, but through its representational destabilization of revolutionary history.

 2. Does it sufficiently engage with relevant methodologies and secondary literature?

 Partially. While it draws from an impressive theoretical lineage—Foucault, Gorgias, Tacitus, Hayden White—it lacks engagement with contemporary scholarship on political fiction and memory studies (e.g., Kohlmann, Perica). It also omits explicit dialogue with key historiographical critiques of poststructuralist historical relativism.

 3. Is the work clearly and cogently presented?

 No. The argument is obfuscated by overly dense prose and a lack of structural signposting. The rhetorical style mirrors the novel’s complexity but often at the expense of scholarly clarity.

 4. Is the argument persuasive and supported by evidence?

 Partially. The close reading is compelling but lacks systematic development. A working definition of “political novel” is missing, and theoretical concepts are frequently left unelaborated, requiring the reader to infer too much.

 5. Are all source data and materials underlying the results available?

 Yes. The article references an extensive and appropriate body of primary and secondary materials.

 6. Does the article contribute to cultural, historical, or social understanding of the field?

 Yes, conditionally. It has the potential to offer a substantial contribution to the study of historical fiction and revolutionary representation. However, its current form limits interdisciplinary accessibility and insufficiently addresses the ideological stakes of its claims.

**Major Recommendations for Revision**

 1. Stylistic and Structural Clarity

- Substantially revise the prose to prioritize clarity, economy, and readability.
- Introduce clear section headings, transitions, and argumentative scaffolding.
- Provide an early summary of the thesis and a conclusive synthesis that clearly outlines the article’s implications.

2. Articulating the Political Novel: Aesthetic, Epistemological, and Ideological Stakes

- Articulate what is meant by “political” in this context: is it formalist, ethical, ideological, aesthetic, or rhetorical?
- Given the article’s emphasis on the fictionalization of political memory and its engagement with the aesthetics of historiography, it would benefit significantly from a more direct and sustained dialogue with the work of Hayden White—particularly Metahistory. White’s foundational arguments regarding the narrative structures underlying historical explanation, the tropological shaping of meaning, and the blurred boundaries between historiography and fiction offer critical conceptual tools that align closely with the article’s approach. Explicitly invoking White would clarify and deepen the article’s theoretical architecture, lending greater coherence to its claims about aesthetic mediation and historical discourse.
- Clarify whether the article is interpreting *The Eleven* as an instance of a 'poststructuralist' political novel, and explicitly acknowledge the critiques leveled by social historians such as Richard Evans, Christopher Lloyd, and Georg Iggers. These scholars challenge the tendency within poststructuralist historiography to conflate epistemological relativism (i.e., that knowledge is always mediated by discourse) with ontological relativism (i.e., that reality itself is reducible to discourse). They also warn of the political consequences of such approaches, particularly the risk of undermining the distinction between historical fact and narrative construction—thereby potentially enabling post-factual revisionist or depoliticizing accounts of historical events.

3. Historiographical Positioning

- The article must better situate *The Eleven* in relation to François Furet’s revisionist historiography, particularly his framing of the French Revolution as inherently totalitarian. While the article claims Michon departs from this model, it does not convincingly demonstrate how or to what extent.
- Michon’s narrative may aesthetically reframe revolutionary violence, but it remains ideologically ambiguous—perhaps even reproducing the pessimistic view of revolutionary teleology advanced by Furet. The article should clearly differentiate Michon’s aesthetic from Furet’s ideological critique of the Revolution as inherently totalitarian.
- Furthermore, the article’s abstract makes the provocative claim that *The Eleven* ultimately serves to "redeem the Revolution" through a literary re-enactment of the "politics of fiction." This assertion—while rich in potential—remains critically underdeveloped in the body of the article. It is never clearly elaborated what is meant by "redemption" in this context: Is the Revolution redeemed aesthetically, philosophically, morally, or politically? Nor is it shown how Michon's fictional apparatus achieves such redemption beyond aestheticizing the traumatic spectacle of revolutionary terror.

4. Engage Counter-Historiographical Perspectives

- Introduce social historians of revolution (e.g., Arno Mayer, Enzo Traverso, Domenico Losurdo) who challenge the equation of revolution and terror, and contextualize the material, social, and political conditions of revolutionary violence.
- The work would also benefit from referencing Kohlmann and Perica, whose framing of literature’s political uses resists the aestheticist flattening of history into allegory. As Perica and Kohlmann note (drawing on Brecht), “literary–political constellations cannot be understood without accounting for their historical, social, and political contexts.” This critical premise stands in contrast to poststructuralist tendencies toward abstraction, which underpin both *The Eleven* and this article’s framing.
- This will help avoid the naturalization of terror as an aesthetic inevitability, a position that risks aligning with counter-revolutionary and anti-communist revisionisms—notably represented in controversial projects like *The Black Book of Communism*.

5. Address the Politics of Representation

- The idea that fiction “liquidates reality” must be critically unpacked. Without contextualization, it risks echoing contemporary post-factual and far-right revisionist narratives that dismiss revolutionary and anti-fascist struggles as ideological fabrications.
- Reference to Valencia-García’s *Far-Right Revisionism and the End of History* can highlight the real-world stakes of historical abstraction and aesthetic depoliticization.
- A critical question emerges: What are the political consequences of conflating narrative, memory, and myth in the age of institutionalized memory politics and post-factual nationalism?

6. Methodological Transparency

- The article lacks an explicit account of its methodological approach. A brief paragraph delineating the use of rhetorical reading, dispositif theory, ekphrastic analysis, or poetics of historiography would greatly benefit interdisciplinary readers.

**Minor Points**

- In-line citations (e.g., “5 on page 13”) disrupt narrative flow. Use standard references or endnotes.
- Define metaphors such as “liquidation of reality”, “page of darkness”, and “rabbit hole” more clearly.
- Distinguish between narrator, implied author, and critical position within the novel to avoid conflation.

** Conclusion**

 In sum, this article reveals the political stakes of historiographic fiction with admirable depth, but its potential is hindered by issues of clarity, theoretical framing, and historiographical engagement. To realize its full potential, the article must more clearly articulate its theoretical framework, enhance accessibility, and critically address the political stakes of its central claims. With such revisions, this article could serve as a landmark contribution to the study of poststructuralist historical fiction, the aesthetics of memory, and the politics of revolutionary representation.

If any, are all the source data and materials underlying the results available?

Yes

Is the work original in terms of material and argument?

Yes

Is the work clearly and cogently presented?

No

Is the argument persuasive and supported by evidence?

Yes

Does the research article contribute to the cultural, historical, social understanding of the field?

Partly

Does it sufficiently engage with relevant methodologies and secondary literature on the topic?

Partly

Reviewer Expertise:

Historiography of the French and Russian Revolutions; Social and Intellectual history; Politics of Memory; Historical Sociology; Philosophy of Social Science, Social Theory, International Relations Theories, Critical Realism.

I confirm that I have read this submission and believe that I have an appropriate level of expertise to confirm that it is of an acceptable scientific standard, however I have significant reservations, as outlined above.

## References

[1] Evans, Richard J. (1997). In Defence of History. London: Granta Books.

[2] Iggers, Georg G. (1997). Historiography in the Twentieth Century: From Scientific Objectivity to the Postmodern Challenge. Middletown: Wesleyan University Press.

[3] Lloyd, Christopher (1993). The Structures of History. Oxford: Blackwell.

[4] Mayer, A. J. (2000). The Furies: Violence and Terror in the French and Russian Revolutions. Princeton University Press.

[5] Traverso, E. (2021). Revolution: An Intellectual History. Verso.

[6] Traverso, E. (2017). Totalitarianism between history and theory. History and Theory, 56, 97–118.

[7] Traverso, E. (2011). L’histoire comme champ de bataille. Interpréter les violences du XXe siècle. Paris: La Découverte.

[8] Losurdo, D. (2015). War and Revolution: Rethinking the Twentieth Century. Verso Books.

[9] White, H. (1973). Metahistory: The Historical Imagination in Nineteenth-Century Europe. Johns Hopkins University Press.

[10] Kohlmann, B., & Perica, I. (Eds.). (2024). The Political Uses of Literature: Global Perspectives and Theoretical Approaches, 1920–2020. New York: Bloomsbury Academic.

[11] Courtois, S., Werth, N., Panné, J.-L., Paczkowski, A., Bartosek, K., & Margolin, J.-L. (1999). The Black Book of Communism: Crimes, Terror, Repression. Harvard University Press.

[12] Behr, V., Blaive, M., Constantin, A., Neumayer, L., & Zombory, M. (2020). “An anti-communist consensus: The Black Book of Communism in Pan-European Perspective.” Revue d'études comparatives Est-Ouest, 51(2), 55–83.

[13] Valencia-García, L. D. (2020). Far-Right Revisionism and the End of History: Alt/Histories. Routledge.

# Reviewer report: Reviewer response for version 1

**DOI:** 10.21956/openreseurope.21550.r55704

**Published:** 2025-08-06

**Authors:** Hüseyin Alhas

**Affiliations:** 1 Social Sciences University of Ankara, Ankara, Turkey

The manuscript asks whether Pierre Michon’s *Les Onze* (2009; *The Eleven*) ought to be considered a political novel. It proceeds by treating the novel’s imagined painting of the Comité de Salut Public as a heuristic device that dramatises the conditions under which revolutionary politics can be shown or narrated. Four analytic axes structure the discussion. First, the essay foregrounds ekphrasis and classical rhetoric. The narrator’s cicerone-like discourse is read as a self-reflexive enactment of Greek *ekphrasis*, transforming an absent canvas into an object of shared vision for characters and readers alike. Secondly, the argument turns to historiography versus fiction. By juxtaposing Jules Michelet’s romantic narrative with François Furet’s revisionism, the author suggests that Michon offers a micro-historical counter-model that exposes the ideological investments of grand revolutionary histories. Thirdly, the article explores Tacitean ambiguity. Drawing on Erich Auerbach’s account of Tacitus, it links Michon’s syntactic obscurity to the ethical indeterminacy of the Terror, demonstrating how linguistic hesitancy mirrors political ambivalence. Finally, the study analyses figural politics. Recurring father–son imagery is interpreted as an allegory of regicide and perpetual symbolic parricide in French cultural memory, thereby revealing how the novel inscribes the Revolution’s unresolved familial trauma. The article concludes that *Les Onze* is political precisely because it redefines politics as the scene in which fiction invents its own conditions of visibility.

 In terms of originality, the dialogue between *ekphrasis*, micro-history, and revolutionary iconography is genuinely innovative. The manuscript brings together classical rhetoric, visual theory, and historiography in a manner that deserves attention. Regarding engagement with secondary literature, the author draws on Michelet, Furet, Auerbach, and studies of micro-history. However, the piece bypasses scholarship on the political novel as a genre. Integrating critical works on this issue would add a necessary layer of generic self-consciousness and situate the argument within broader debates. This is quite important and will better the article dramatically. As for clarity of presentation, the prose is generally elegant and precise, though the core thesis emerges only in the closing pages. I recommend inserting a stand-alone thesis paragraph at the end of the introduction. Finally, the study contributes meaningfully to cultural and historical understanding. By reframing the Terror as a crisis of representation, it invites fresh reflection on French revolutionary memory and prompts a reassessment of the boundaries of political fiction. As the manuscript observes at its most incisive—“The failure of literature is always the gain of literature”—Michon’s text leverages its own fictional status to interrogate how history is staged, seen, and believed. Thus, this study makes a valuable contribution, but a revised version that clarifies its thesis, broadens its engagement with genre theory, and streamlines its structure would more fully realise its intellectual promise.

If any, are all the source data and materials underlying the results available?

No source data required

Is the work original in terms of material and argument?

Yes

Is the work clearly and cogently presented?

Yes

Is the argument persuasive and supported by evidence?

Yes

Does the research article contribute to the cultural, historical, social understanding of the field?

Yes

Does it sufficiently engage with relevant methodologies and secondary literature on the topic?

Partly

Reviewer Expertise:

Cultural materialism, the interplay of politics and literature, Epic poetry, the literature and political culture of the French Revolution, Romanticism

I confirm that I have read this submission and believe that I have an appropriate level of expertise to confirm that it is of an acceptable scientific standard, however I have significant reservations, as outlined above.
